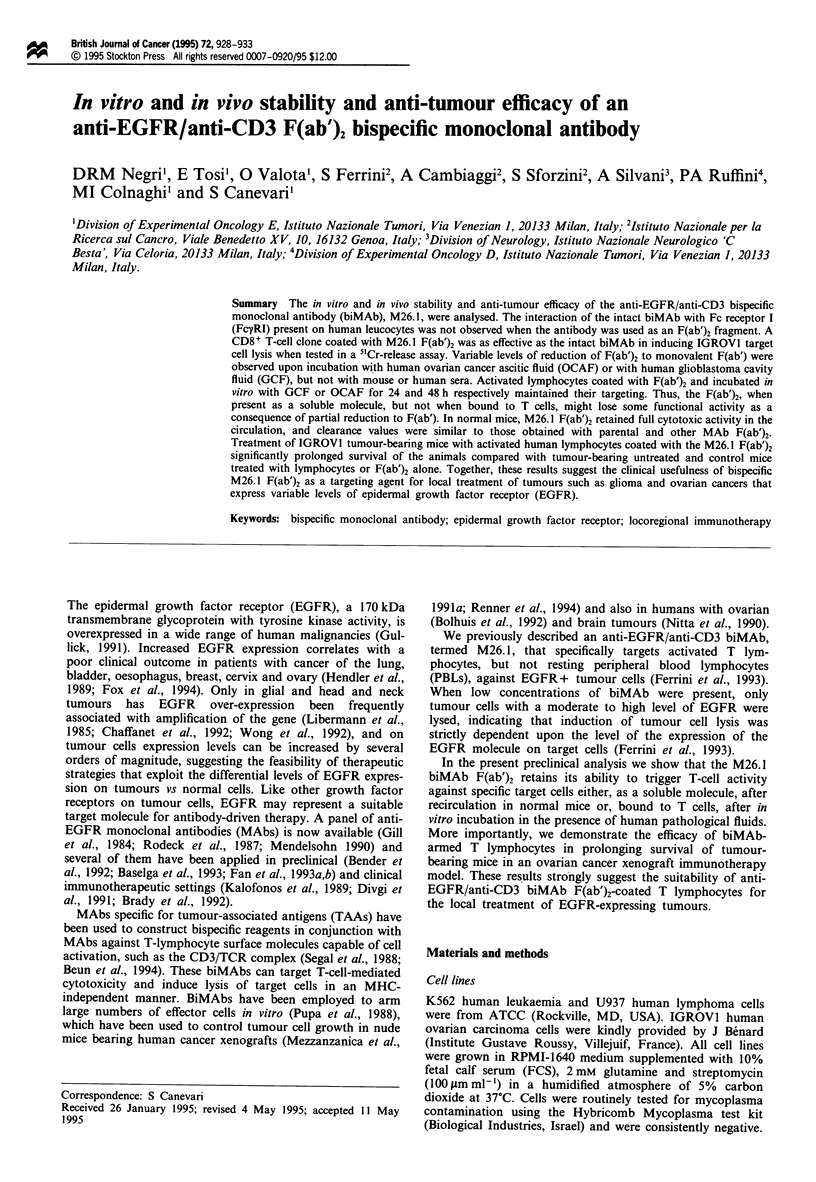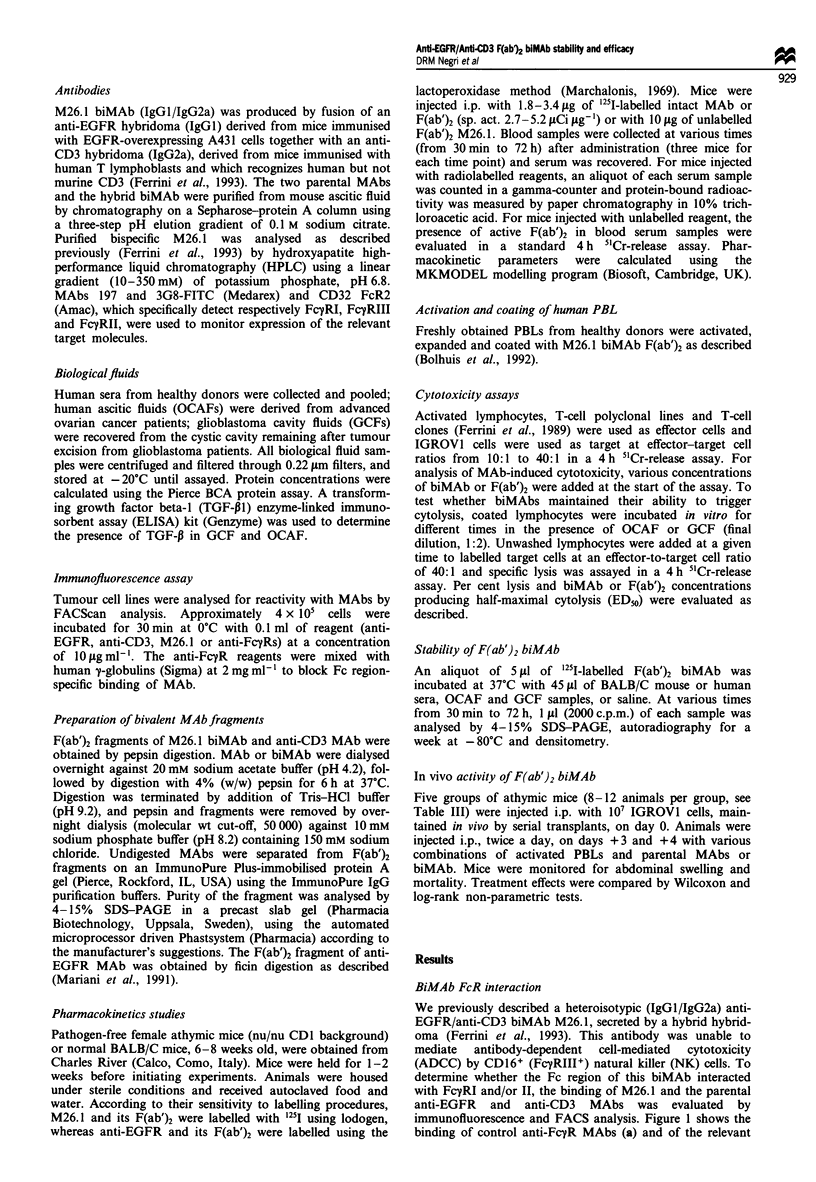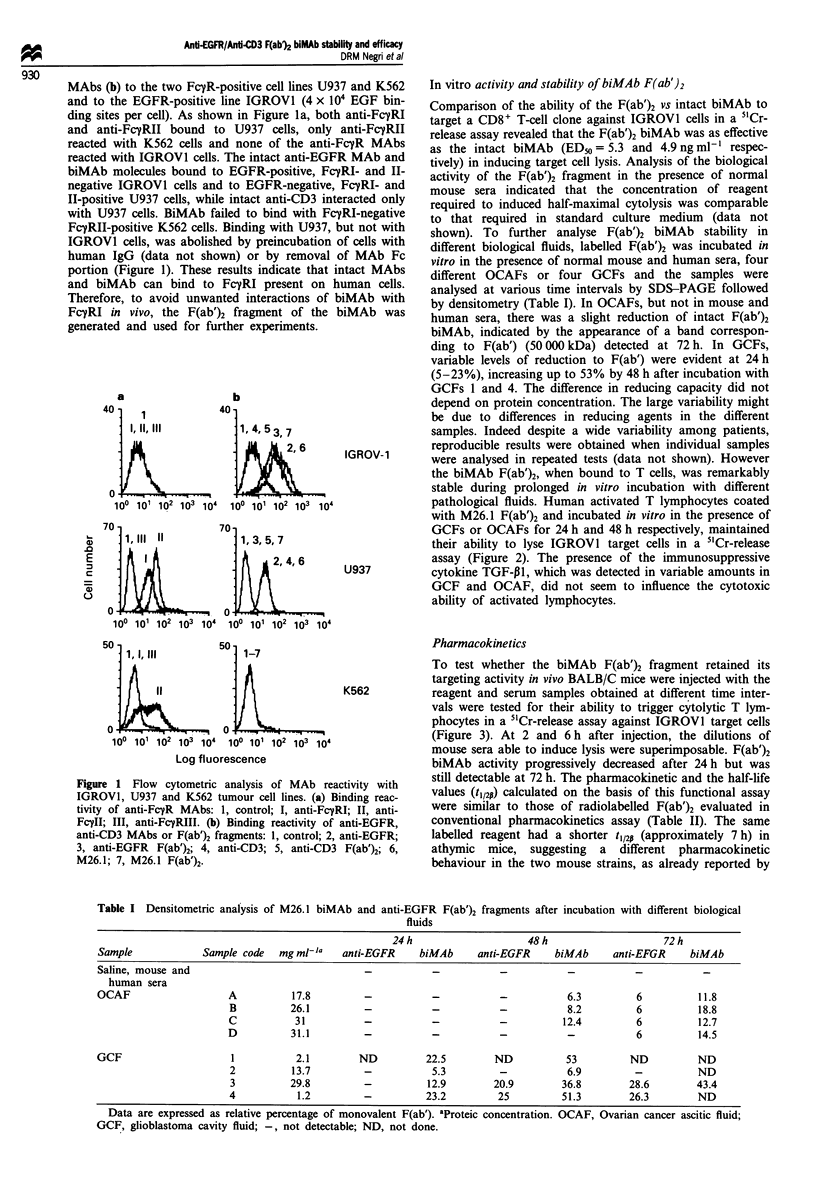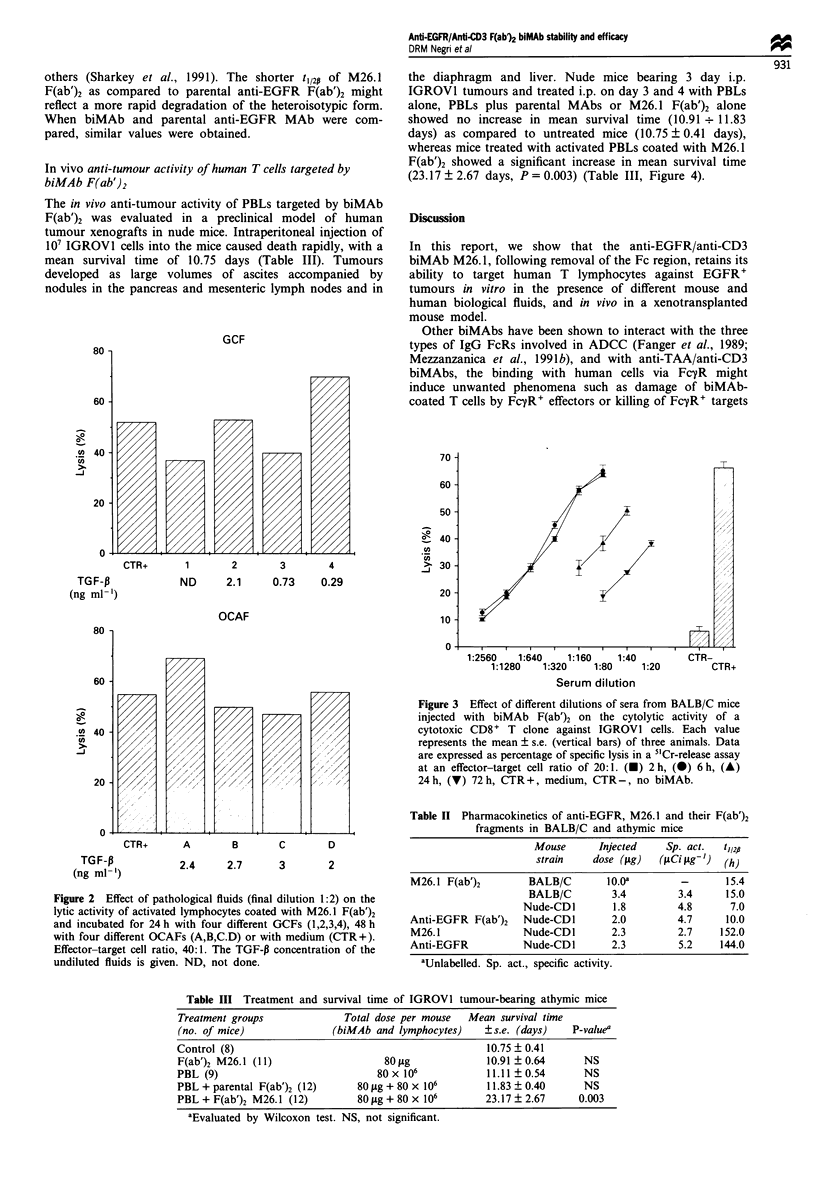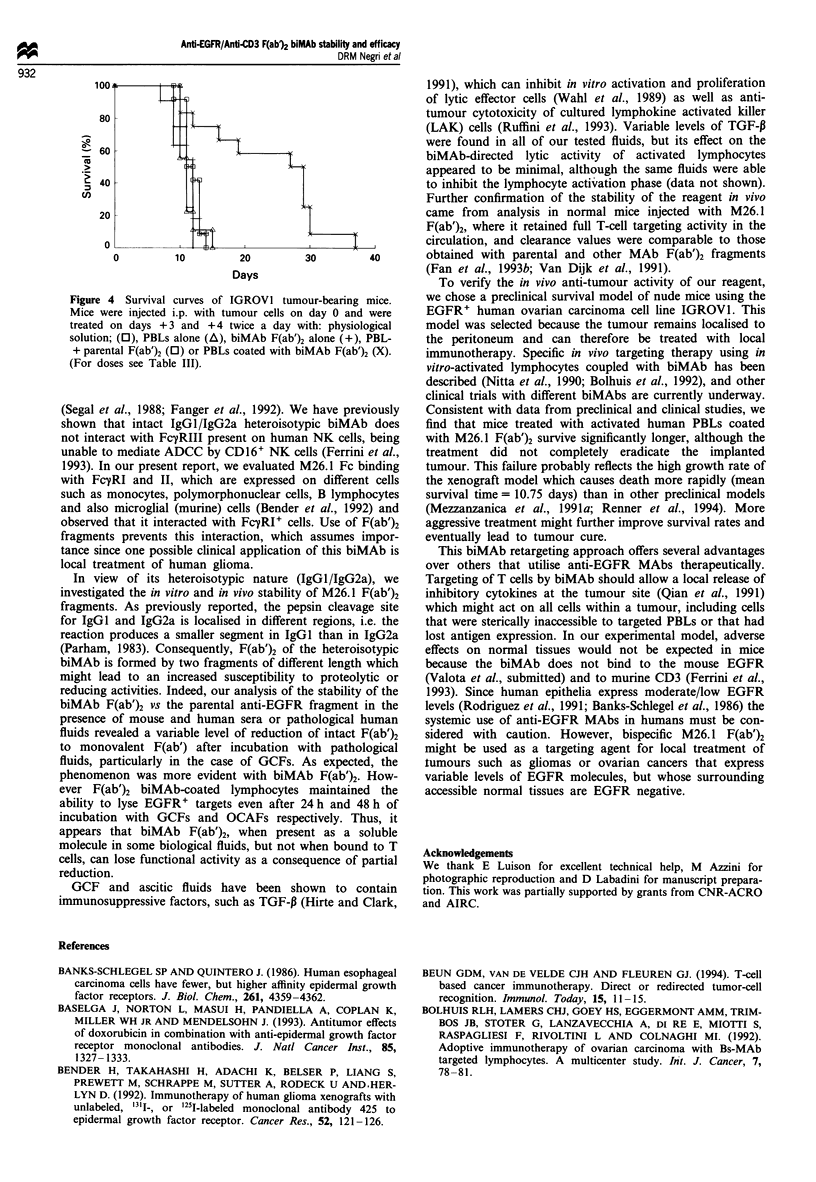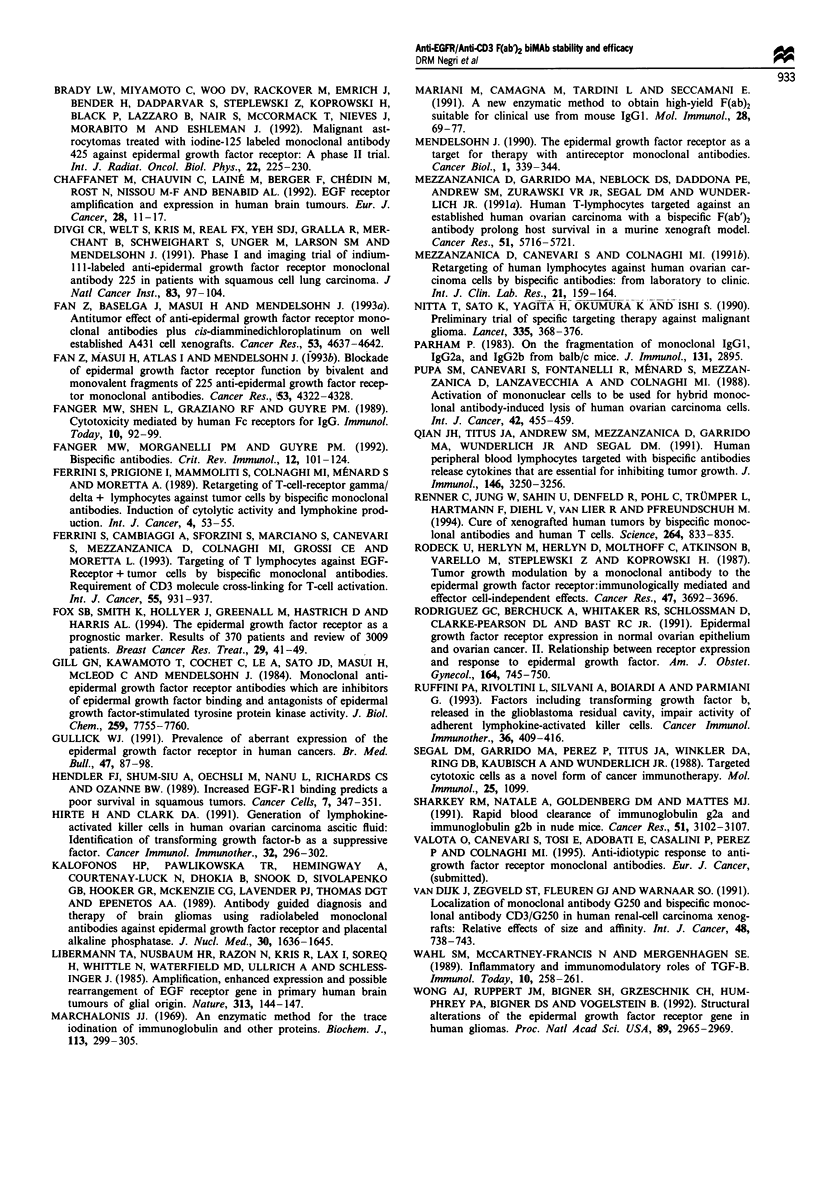# In vitro and in vivo stability and anti-tumour efficacy of an anti-EGFR/anti-CD3 F(ab')2 bispecific monoclonal antibody.

**DOI:** 10.1038/bjc.1995.435

**Published:** 1995-10

**Authors:** D. R. Negri, E. Tosi, O. Valota, S. Ferrini, A. Cambiaggi, S. Sforzini, A. Silvani, P. A. Ruffini, M. I. Colnaghi, S. Canevari

**Affiliations:** Division of Experimental Oncology E, Istituto Nazionale Tumori, Milan, Italy.

## Abstract

The in vitro and in vivo stability and anti-tumour efficacy of the anti-EGFR/anti-CD3 bispecific monoclonal antibody (biMAb), M26.1, were analysed. The interaction of the intact biMAb with Fc receptor I (Fc gamma RI) present on human leucocytes was not observed when the antibody was used as an F(ab')2 fragment. A CD8+ T-cell clone coated with M26.1 F(ab')2 was as effective as the intact biMAb in inducing IGROV1 target cell lysis when tested in a 51Cr-release assay. Variable levels of reduction of F(ab')2 to monovalent F(ab') were observed upon incubation with human ovarian cancer ascitic fluid (OCAF) or with human glioblastoma cavity fluid (GCF), but not with mouse or human sera. Activated lymphocytes coated with F(ab')2 and incubated in vitro with GCF or OCAF for 24 and 48 h respectively maintained their targeting. Thus, the F(ab')2, when present as a soluble molecule, but not when bound to T cells, might lose some functional activity as a consequence of partial reduction to F(ab'). In normal mice, M26.1 F(ab')2 retained full cytotoxic activity in the circulation, and clearance values were similar to those obtained with parental and other MAb F(ab')2. Treatment of IGROV1 tumour-bearing mice with activated human lymphocytes coated with the M26.1 F(ab')2 significantly prolonged survival of the animals compared with tumour-bearing untreated and control mice treated with lymphocytes or F(ab')2 alone. Together, these results suggest the clinical usefulness of bispecific M26.1 F(ab')2 as a targeting agent for local treatment of tumours such as glioma and ovarian cancers that express variable levels of epidermal growth factor receptor (EGFR).


British Journal of Cancer (1995) 72, 928–933
© 1995 Stockton Press All rights reserved 0007–0920/95 $12.00

### *In vitro* and *in vivo* stability and anti-tumour efficacy of an anti-EGFR/anti-CD3 F(ab')$_2$ bispecific monoclonal antibody

DRM Negri[1], E Tosi[1], O Valota[1], S Ferrini[2], A Cambiaggi[2], S Sforzini[2], A Silvani[3], PA Ruffini[4], MI Colnaghi[1] and S Canevari[1]

[1]*Division of Experimental Oncology E, Istituto Nazionale Tumori, Via Venezian 1, 20133 Milan, Italy;* [2]*Istituto Nazionale per la Ricerca sul Cancro, Viale Benedetto XV, 10, 16132 Genoa, Italy;* [3]*Division of Neurology, Istituto Nazionale Neurologico 'C Besta', Via Celoria, 20133 Milan, Italy;* [4]*Division of Experimental Oncology D, Istituto Nazionale Tumori, Via Venezian 1, 20133 Milan, Italy.*

**Summary** The *in vitro* and *in vivo* stability and anti-tumour efficacy of the anti-EGFR/anti-CD3 bispecific monoclonal antibody (biMAb), M26.1, were analysed. The interaction of the intact biMAb with Fc receptor I (FcγRI) present on human leucocytes was not observed when the antibody was used as an F(ab')$_2$ fragment. A $CD8^+$ T-cell clone coated with M26.1 F(ab')$_2$ was as effective as the intact biMAb in inducing IGROV1 target cell lysis when tested in a $^{51}Cr$-release assay. Variable levels of reduction of F(ab')$_2$ to monovalent F(ab') were observed upon incubation with human ovarian cancer ascitic fluid (OCAF) or with human glioblastoma cavity fluid (GCF), but not with mouse or human sera. Activated lymphocytes coated with F(ab')$_2$ and incubated *in vitro* with GCF or OCAF for 24 and 48 h respectively maintained their targeting. Thus, the F(ab')$_2$, when present as a soluble molecule, but not when bound to T cells, might lose some functional activity as a consequence of partial reduction to F(ab'). In normal mice, M26.1 F(ab')$_2$ retained full cytotoxic activity in the circulation, and clearance values were similar to those obtained with parental and other MAb F(ab')$_2$. Treatment of IGROV1 tumour-bearing mice with activated human lymphocytes coated with the M26.1 F(ab')$_2$ significantly prolonged survival of the animals compared with tumour-bearing untreated and control mice treated with lymphocytes or F(ab')$_2$ alone. Together, these results suggest the clinical usefulness of bispecific M26.1 F(ab')$_2$ as a targeting agent for local treatment of tumours such as glioma and ovarian cancers that express variable levels of epidermal growth factor receptor (EGFR).

**Keywords:** bispecific monoclonal antibody; epidermal growth factor receptor; locoregional immunotherapy

The epidermal growth factor receptor (EGFR), a 170 kDa transmembrane glycoprotein with tyrosine kinase activity, is overexpressed in a wide range of human malignancies (Gullick, 1991). Increased EGFR expression correlates with a poor clinical outcome in patients with cancer of the lung, bladder, oesophagus, breast, cervix and ovary (Hendler *et al.*, 1989; Fox *et al.*, 1994). Only in glial and head and neck tumours has EGFR over-expression been frequently associated with amplification of the gene (Libermann *et al.*, 1985; Chaffanet *et al.*, 1992; Wong *et al.*, 1992), and on tumour cells expression levels can be increased by several orders of magnitude, suggesting the feasibility of therapeutic strategies that exploit the differential levels of EGFR expression on tumours *vs* normal cells. Like other growth factor receptors on tumour cells, EGFR may represent a suitable target molecule for antibody-driven therapy. A panel of anti-EGFR monoclonal antibodies (MAbs) is now available (Gill *et al.*, 1984; Rodeck *et al.*, 1987; Mendelsohn 1990) and several of them have been applied in preclinical (Bender *et al.*, 1992; Baselga *et al.*, 1993; Fan *et al.*, 1993*a,b*) and clinical immunotherapeutic settings (Kalofonos *et al.*, 1989; Divgi *et al.*, 1991; Brady *et al.*, 1992).

MAbs specific for tumour-associated antigens (TAAs) have been used to construct bispecific reagents in conjunction with MAbs against T-lymphocyte surface molecules capable of cell activation, such as the CD3/TCR complex (Segal *et al.*, 1988; Beun *et al.*, 1994). These biMAbs can target T-cell-mediated cytotoxicity and induce lysis of target cells in an MHC-independent manner. BiMAbs have been employed to arm large numbers of effector cells *in vitro* (Pupa *et al.*, 1988), which have been used to control tumour cell growth in nude mice bearing human cancer xenografts (Mezzanzanica *et al.*, 1991*a*; Renner *et al.*, 1994) and also in humans with ovarian (Bolhuis *et al.*, 1992) and brain tumours (Nitta *et al.*, 1990).

We previously described an anti-EGFR/anti-CD3 biMAb, termed M26.1, that specifically targets activated T lymphocytes, but not resting peripheral blood lymphocytes (PBLs), against EGFR+ tumour cells (Ferrini *et al.*, 1993). When low concentrations of biMAb were present, only tumour cells with a moderate to high level of EGFR were lysed, indicating that induction of tumour cell lysis was strictly dependent upon the level of the expression of the EGFR molecule on target cells (Ferrini *et al.*, 1993).

In the present preclinical analysis we show that the M26.1 biMAb F(ab')$_2$ retains its ability to trigger T-cell activity against specific target cells either, as a soluble molecule, after recirculation in normal mice or, bound to T cells, after *in vitro* incubation in the presence of human pathological fluids. More importantly, we demonstrate the efficacy of biMAb-armed T lymphocytes in prolonging survival of tumour-bearing mice in an ovarian cancer xenograft immunotherapy model. These results strongly suggest the suitability of anti-EGFR/anti-CD3 biMAb F(ab')$_2$-coated T lymphocytes for the local treatment of EGFR-expressing tumours.

### Materials and methods

### *Cell lines*

K562 human leukaemia and U937 human lymphoma cells were from ATCC (Rockville, MD, USA). IGROV1 human ovarian carcinoma cells were kindly provided by J Bénard (Institute Gustave Roussy, Villejuif, France). All cell lines were grown in RPMI-1640 medium supplemented with 10% fetal calf serum (FCS), 2 mM glutamine and streptomycin (100 μm ml$^{-1}$) in a humidified atmosphere of 5% carbon dioxide at 37°C. Cells were routinely tested for mycoplasma contamination using the Hybricomb Mycoplasma test kit (Biological Industries, Israel) and were consistently negative.

Correspondence: S Canevari
Received 26 January 1995; revised 4 May 1995; accepted 11 May 1995

*Antibodies*

M26.1 biMAb (IgG1/IgG2a) was produced by fusion of an anti-EGFR hybridoma (IgG1) derived from mice immunised with EGFR-overexpressing A431 cells together with an anti-CD3 hybridoma (IgG2a), derived from mice immunised with human T lymphoblasts and which recognizes human but not murine CD3 (Ferrini *et al.*, 1993). The two parental MAbs and the hybrid biMAb were purified from mouse ascitic fluid by chromatography on a Sepharose–protein A column using a three-step pH elution gradient of 0.1 M sodium citrate. Purified bispecific M26.1 was analysed as described previously (Ferrini *et al.*, 1993) by hydroxyapatite high-performance liquid chromatography (HPLC) using a linear gradient (10–350 mM) of potassium phosphate, pH 6.8. MAbs 197 and 3G8-FITC (Medarex) and CD32 FcR2 (Amac), which specifically detect respectively FcγRI, FcγRIII and FcγRII, were used to monitor expression of the relevant target molecules.

*Biological fluids*

Human sera from healthy donors were collected and pooled; human ascitic fluids (OCAFs) were derived from advanced ovarian cancer patients; glioblastoma cavity fluids (GCFs) were recovered from the cystic cavity remaining after tumour excision from glioblastoma patients. All biological fluid samples were centrifuged and filtered through 0.22 μm filters, and stored at −20°C until assayed. Protein concentrations were calculated using the Pierce BCA protein assay. A transforming growth factor beta-1 (TGF-β1) enzyme-linked immunosorbent assay (ELISA) kit (Genzyme) was used to determine the presence of TGF-β in GCF and OCAF.

*Immunofluorescence assay*

Tumour cell lines were analysed for reactivity with MAbs by FACScan analysis. Approximately $4 \times 10^5$ cells were incubated for 30 min at 0°C with 0.1 ml of reagent (anti-EGFR, anti-CD3, M26.1 or anti-FcγRs) at a concentration of 10 μg ml$^{-1}$. The anti-FcγR reagents were mixed with human γ-globulins (Sigma) at 2 mg ml$^{-1}$ to block Fc region-specific binding of MAb.

*Preparation of bivalent MAb fragments*

F(ab')$_2$ fragments of M26.1 biMAb and anti-CD3 MAb were obtained by pepsin digestion. MAb or biMAb were dialysed overnight against 20 mM sodium acetate buffer (pH 4.2), followed by digestion with 4% (w/w) pepsin for 6 h at 37°C. Digestion was terminated by addition of Tris–HCl buffer (pH 9.2), and pepsin and fragments were removed by overnight dialysis (molecular wt cut-off, 50 000) against 10 mM sodium phosphate buffer (pH 8.2) containing 150 mM sodium chloride. Undigested MAbs were separated from F(ab')$_2$ fragments on an ImmunoPure Plus-immobilised protein A gel (Pierce, Rockford, IL, USA) using the ImmunoPure IgG purification buffers. Purity of the fragment was analysed by 4–15% SDS–PAGE in a precast slab gel (Pharmacia Biotechnology, Uppsala, Sweden), using the automated microprocessor driven Phastsystem (Pharmacia) according to the manufacturer's suggestions. The F(ab')$_2$ fragment of anti-EGFR MAb was obtained by ficin digestion as described (Mariani *et al.*, 1991).

*Pharmacokinetics studies*

Pathogen-free female athymic mice (nu/nu CD1 background) or normal BALB/C mice, 6–8 weeks old, were obtained from Charles River (Calco, Como, Italy). Mice were held for 1–2 weeks before initiating experiments. Animals were housed under sterile conditions and received autoclaved food and water. According to their sensitivity to labelling procedures, M26.1 and its F(ab')$_2$ were labelled with $^{125}$I using Iodogen, whereas anti-EGFR and its F(ab')$_2$ were labelled using the lactoperoxidase method (Marchalonis, 1969). Mice were injected i.p. with 1.8–3.4 μg of $^{125}$I-labelled intact MAb or F(ab')$_2$ (sp. act. 2.7–5.2 μCi μg$^{-1}$) or with 10 μg of unlabelled F(ab')$_2$ M26.1. Blood samples were collected at various times (from 30 min to 72 h) after administration (three mice for each time point) and serum was recovered. For mice injected with radiolabelled reagents, an aliquot of each serum sample was counted in a gamma-counter and protein-bound radioactivity was measured by paper chromatography in 10% trichloroacetic acid. For mice injected with unlabelled reagent, the presence of active F(ab')$_2$ in blood serum samples were evaluated in a standard 4 h $^{51}$Cr-release assay. Pharmacokinetic parameters were calculated using the MKMODEL modelling program (Biosoft, Cambridge, UK).

*Activation and coating of human PBL*

Freshly obtained PBLs from healthy donors were activated, expanded and coated with M26.1 biMAb F(ab')$_2$ as described (Bolhuis *et al.*, 1992).

*Cytotoxicity assays*

Activated lymphocytes, T-cell polyclonal lines and T-cell clones (Ferrini *et al.*, 1989) were used as effector cells and IGROV1 cells were used as target at effector–target cell ratios from 10:1 to 40:1 in a 4 h $^{51}$Cr-release assay. For analysis of MAb-induced cytotoxicity, various concentrations of biMAb or F(ab')$_2$ were added at the start of the assay. To test whether biMAbs maintained their ability to trigger cytolysis, coated lymphocytes were incubated *in vitro* for different times in the presence of OCAF or GCF (final dilution, 1:2). Unwashed lymphocytes were added at a given time to labelled target cells at an effector-to-target cell ratio of 40:1 and specific lysis was assayed in a 4 h $^{51}$Cr-release assay. Per cent lysis and biMAb or F(ab')$_2$ concentrations producing half-maximal cytolysis ($ED_{50}$) were evaluated as described.

*Stability of F(ab')$_2$ biMAb*

An aliquot of 5 μl of $^{125}$I-labelled F(ab')$_2$ biMAb was incubated at 37°C with 45 μl of BALB/C mouse or human sera, OCAF and GCF samples, or saline. At various times from 30 min to 72 h, 1 μl (2000 c.p.m.) of each sample was analysed by 4–15% SDS–PAGE, autoradiography for a week at −80°C and densitometry.

In vivo *activity of F(ab')$_2$ biMAb*

Five groups of athymic mice (8–12 animals per group, see Table III) were injected i.p. with $10^7$ IGROV1 cells, maintained *in vivo* by serial transplants, on day 0. Animals were injected i.p., twice a day, on days +3 and +4 with various combinations of activated PBLs and parental MAbs or biMAb. Mice were monitored for abdominal swelling and mortality. Treatment effects were compared by Wilcoxon and log-rank non-parametric tests.

### Results

*BiMAb FcR interaction*

We previously described a heteroisotypic (IgG1/IgG2a) anti-EGFR/anti-CD3 biMAb M26.1, secreted by a hybrid hybridoma (Ferrini *et al.*, 1993). This antibody was unable to mediate antibody-dependent cell-mediated cytotoxicity (ADCC) by CD16$^+$ (FcγRIII$^+$) natural killer (NK) cells. To determine whether the Fc region of this biMAb interacted with FcγRI and/or II, the binding of M26.1 and the parental anti-EGFR and anti-CD3 MAbs was evaluated by immunofluorescence and FACS analysis. Figure 1 shows the binding of control anti-FcγR MAbs (**a**) and of the relevant

MAbs (**b**) to the two FcγR-positive cell lines U937 and K562 and to the EGFR-positive line IGROV1 ($4 \times 10^4$ EGF binding sites per cell). As shown in Figure 1a, both anti-FcγRI and anti-FcγRII bound to U937 cells, only anti-FcγRII reacted with K562 cells and none of the anti-FcγR MAbs reacted with IGROV1 cells. The intact anti-EGFR MAb and biMAb molecules bound to EGFR-positive, FcγRI- and II-negative IGROV1 cells and to EGFR-negative, FcγRI- and II-positive U937 cells, while intact anti-CD3 interacted only with U937 cells. BiMAb failed to bind with FcγRI-negative FcγRII-positive K562 cells. Binding with U937, but not with IGROV1 cells, was abolished by preincubation of cells with human IgG (data not shown) or by removal of MAb Fc portion (Figure 1). These results indicate that intact MAbs and biMAb can bind to FcγRI present on human cells. Therefore, to avoid unwanted interactions of biMAb with FcγRI *in vivo*, the F(ab')$_2$ fragment of the biMAb was generated and used for further experiments.

**Figure 1** Flow cytometric analysis of MAb reactivity with IGROV1, U937 and K562 tumour cell lines. (**a**) Binding reactivity of anti-FcγR MAbs: 1, control; I, anti-FcγRI; II, anti-FcγII; III, anti-FcγRIII. (**b**) Binding reactivity of anti-EGFR, anti-CD3 MAbs or F(ab')$_2$ fragments: 1, control; 2, anti-EGFR; 3, anti-EGFR F(ab')$_2$; 4, anti-CD3; 5, anti-CD3 F(ab')$_2$; 6, M26.1; 7, M26.1 F(ab')$_2$.

### In vitro *activity and stability of biMAb F(ab')$_2$*

Comparison of the ability of the F(ab')$_2$ *vs* intact biMAb to target a $CD8^+$ T-cell clone against IGROV1 cells in a $^{51}$Cr-release assay revealed that the F(ab')$_2$ biMAb was as effective as the intact biMAb ($ED_{50} = 5.3$ and $4.9\ ng\ ml^{-1}$ respectively) in inducing target cell lysis. Analysis of the biological activity of the F(ab')$_2$ fragment in the presence of normal mouse sera indicated that the concentration of reagent required to induced half-maximal cytolysis was comparable to that required in standard culture medium (data not shown). To further analyse F(ab')$_2$ biMAb stability in different biological fluids, labelled F(ab')$_2$ was incubated *in vitro* in the presence of normal mouse and human sera, four different OCAFs or four GCFs and the samples were analysed at various time intervals by SDS–PAGE followed by densitometry (Table I). In OCAFs, but not in mouse and human sera, there was a slight reduction of intact F(ab')$_2$ biMAb, indicated by the appearance of a band corresponding to F(ab') (50 000 kDa) detected at 72 h. In GCFs, variable levels of reduction to F(ab') were evident at 24 h (5–23%), increasing up to 53% by 48 h after incubation with GCFs 1 and 4. The difference in reducing capacity did not depend on protein concentration. The large variability might be due to differences in reducing agents in the different samples. Indeed despite a wide variability among patients, reproducible results were obtained when individual samples were analysed in repeated tests (data not shown). However the biMAb F(ab')$_2$, when bound to T cells, was remarkably stable during prolonged *in vitro* incubation with different pathological fluids. Human activated T lymphocytes coated with M26.1 F(ab')$_2$ and incubated *in vitro* in the presence of GCFs or OCAFs for 24 h and 48 h respectively, maintained their ability to lyse IGROV1 target cells in a $^{51}$Cr-release assay (Figure 2). The presence of the immunosuppressive cytokine TGF-β1, which was detected in variable amounts in GCF and OCAF, did not seem to influence the cytotoxic ability of activated lymphocytes.

### *Pharmacokinetics*

To test whether the biMAb F(ab')$_2$ fragment retained its targeting activity *in vivo* BALB/C mice were injected with the reagent and serum samples obtained at different time intervals were tested for their ability to trigger cytolytic T lymphocytes in a $^{51}$Cr-release assay against IGROV1 target cells (Figure 3). At 2 and 6 h after injection, the dilutions of mouse sera able to induce lysis were superimposable. F(ab')$_2$ biMAb activity progressively decreased after 24 h but was still detectable at 72 h. The pharmacokinetic and the half-life values ($t_{1/2\beta}$) calculated on the basis of this functional assay were similar to those of radiolabelled F(ab')$_2$ evaluated in conventional pharmacokinetics assay (Table II). The same labelled reagent had a shorter $t_{1/2\beta}$ (approximately 7 h) in athymic mice, suggesting a different pharmacokinetic behaviour in the two mouse strains, as already reported by

**Table I** Densitometric analysis of M26.1 biMAb and anti-EGFR F(ab')$_2$ fragments after incubation with different biological fluids

| | | | *24 h* | | *48 h* | | *72 h* | |
|---|---|---|---|---|---|---|---|---|
| *Sample* | *Sample code* | *mg ml$^{-1a}$* | *anti-EGFR* | *biMAb* | *anti-EGFR* | *biMAb* | *anti-EFGR* | *biMAb* |
| Saline, mouse and human sera | | | – | – | – | – | – | – |
| OCAF | A | 17.8 | – | – | – | 6.3 | 6 | 11.8 |
| | B | 26.1 | – | – | – | 8.2 | 6 | 18.8 |
| | C | 31 | – | – | – | 12.4 | 6 | 12.7 |
| | D | 31.1 | – | – | – | – | 6 | 14.5 |
| GCF | 1 | 2.1 | ND | 22.5 | ND | 53 | ND | ND |
| | 2 | 13.7 | – | 5.3 | – | 6.9 | – | ND |
| | 3 | 29.8 | – | 12.9 | 20.9 | 36.8 | 28.6 | 43.4 |
| | 4 | 1.2 | – | 23.2 | 25 | 51.3 | 26.3 | ND |

Data are expressed as relative percentage of monovalent F(ab'). [a]Proteic concentration. OCAF, Ovarian cancer ascitic fluid; GCF, glioblastoma cavity fluid; –, not detectable; ND, not done.

others (Sharkey *et al.*, 1991). The shorter $t_{1/2\beta}$ of M26.1 F(ab')$_2$ as compared to parental anti-EGFR F(ab')$_2$ might reflect a more rapid degradation of the heteroisotypic form. When biMAb and parental anti-EGFR MAb were compared, similar values were obtained.

### In vivo *anti-tumour activity of human T cells targeted by biMAb F(ab')$_2$*

The *in vivo* anti-tumour activity of PBLs targeted by biMAb F(ab')$_2$ was evaluated in a preclinical model of human tumour xenografts in nude mice. Intraperitoneal injection of $10^7$ IGROV1 cells into the mice caused death rapidly, with a mean survival time of 10.75 days (Table III). Tumours developed as large volumes of ascites accompanied by nodules in the pancreas and mesenteric lymph nodes and in the diaphragm and liver. Nude mice bearing 3 day i.p. IGROV1 tumours and treated i.p. on day 3 and 4 with PBLs alone, PBLs plus parental MAbs or M26.1 F(ab')$_2$ alone showed no increase in mean survival time (10.91 ÷ 11.83 days) as compared to untreated mice (10.75 ± 0.41 days), whereas mice treated with activated PBLs coated with M26.1 F(ab')$_2$ showed a significant increase in mean survival time (23.17 ± 2.67 days, $P = 0.003$) (Table III, Figure 4).

**Figure 2** Effect of pathological fluids (final dilution 1:2) on the lytic activity of activated lymphocytes coated with M26.1 F(ab')$_2$ and incubated for 24 h with four different GCFs (1,2,3,4), 48 h with four different OCAFs (A,B,C.D) or with medium (CTR+). Effector–target cell ratio, 40:1. The TGF-β concentration of the undiluted fluids is given. ND, not done.

### Discussion

In this report, we show that the anti-EGFR/anti-CD3 biMAb M26.1, following removal of the Fc region, retains its ability to target human T lymphocytes against EGFR$^+$ tumours *in vitro* in the presence of different mouse and human biological fluids, and *in vivo* in a xenotransplanted mouse model.

Other biMAbs have been shown to interact with the three types of IgG FcRs involved in ADCC (Fanger *et al.*, 1989; Mezzanzanica *et al.*, 1991*b*), and with anti-TAA/anti-CD3 biMAbs, the binding with human cells via FcγR might induce unwanted phenomena such as damage of biMAb-coated T cells by FcγR$^+$ effectors or killing of FcγR$^+$ targets

**Figure 3** Effect of different dilutions of sera from BALB/C mice injected with biMAb F(ab')$_2$ on the cytolytic activity of a cytotoxic CD8$^+$ T clone against IGROV1 cells. Each value represents the mean ± s.e. (vertical bars) of three animals. Data are expressed as percentage of specific lysis in a $^{51}$Cr-release assay at an effector–target cell ratio of 20:1. (■) 2 h, (●) 6 h, (▲) 24 h, (▼) 72 h, CTR+, medium, CTR–, no biMAb.

**Table II** Pharmacokinetics of anti-EGFR, M26.1 and their F(ab')$_2$ fragments in BALB/C and athymic mice

| | *Mouse strain* | *Injected dose (µg)* | *Sp. act. (µCi µg$^{-1}$)* | $t_{1/2\beta}$ *(h)* |
|---|---|---|---|---|
| M26.1 F(ab')$_2$ | BALB/C | 10.0[a] | – | 15.4 |
| | BALB/C | 3.4 | 3.4 | 15.0 |
| | Nude-CD1 | 1.8 | 4.8 | 7.0 |
| Anti-EGFR F(ab')$_2$ | Nude-CD1 | 2.0 | 4.7 | 10.0 |
| M26.1 | Nude-CD1 | 2.3 | 2.7 | 152.0 |
| Anti-EGFR | Nude-CD1 | 2.3 | 5.2 | 144.0 |

[a]Unlabelled. Sp. act., specific activity.

**Table III** Treatment and survival time of IGROV1 tumour-bearing athymic mice

| *Treatment groups (no. of mice)* | *Total dose per mouse (biMAb and lymphocytes)* | *Mean survival time ± s.e. (days)* | P-*value*[a] |
|---|---|---|---|
| Control (8) | | 10.75 ± 0.41 | |
| F(ab')$_2$ M26.1 (11) | 80 µg | 10.91 ± 0.64 | NS |
| PBL (9) | 80 × $10^6$ | 11.11 ± 0.54 | NS |
| PBL + parental F(ab')$_2$ (12) | 80 µg + 80 × $10^6$ | 11.83 ± 0.40 | NS |
| PBL + F(ab')$_2$ M26.1 (12) | 80 µg + 80 × $10^6$ | 23.17 ± 2.67 | 0.003 |

[a]Evaluated by Wilcoxon test. NS, not significant.

**Figure 4** Survival curves of IGROV1 tumour-bearing mice. Mice were injected i.p. with tumour cells on day 0 and were treated on days +3 and +4 twice a day with: physiological solution; (□), PBLs alone (Δ), biMAb $F(ab')_2$ alone (+), PBL- + parental $F(ab')_2$ (□) or PBLs coated with biMAb $F(ab')_2$ (X). (For doses see Table III).

(Segal *et al.*, 1988; Fanger *et al.*, 1992). We have previously shown that intact IgG1/IgG2a heteroisotypic biMAb does not interact with FcγRIII present on human NK cells, being unable to mediate ADCC by $CD16^+$ NK cells (Ferrini *et al.*, 1993). In our present report, we evaluated M26.1 Fc binding with FcγRI and II, which are expressed on different cells such as monocytes, polymorphonuclear cells, B lymphocytes and also microglial (murine) cells (Bender *et al.*, 1992) and observed that it interacted with $Fc\gamma RI^+$ cells. Use of $F(ab')_2$ fragments prevents this interaction, which assumes importance since one possible clinical application of this biMAb is local treatment of human glioma.

In view of its heteroisotypic nature (IgG1/IgG2a), we investigated the *in vitro* and *in vivo* stability of M26.1 $F(ab')_2$ fragments. As previously reported, the pepsin cleavage site for IgG1 and IgG2a is localised in different regions, i.e. the reaction produces a smaller segment in IgG1 than in IgG2a (Parham, 1983). Consequently, $F(ab')_2$ of the heteroisotypic biMAb is formed by two fragments of different length which might lead to an increased susceptibility to proteolytic or reducing activities. Indeed, our analysis of the stability of the biMAb $F(ab')_2$ *vs* the parental anti-EGFR fragment in the presence of mouse and human sera or pathological human fluids revealed a variable level of reduction of intact $F(ab')_2$ to monovalent F(ab') after incubation with pathological fluids, particularly in the case of GCFs. As expected, the phenomenon was more evident with biMAb $F(ab')_2$. However $F(ab')_2$ biMAb-coated lymphocytes maintained the ability to lyse $EGFR^+$ targets even after 24 h and 48 h of incubation with GCFs and OCAFs respectively. Thus, it appears that biMAb $F(ab')_2$, when present as a soluble molecule in some biological fluids, but not when bound to T cells, can lose functional activity as a consequence of partial reduction.

GCF and ascitic fluids have been shown to contain immunosuppressive factors, such as TGF-β (Hirte and Clark, 1991), which can inhibit *in vitro* activation and proliferation of lytic effector cells (Wahl *et al.*, 1989) as well as anti-tumour cytotoxicity of cultured lymphokine activated killer (LAK) cells (Ruffini *et al.*, 1993). Variable levels of TGF-β were found in all of our tested fluids, but its effect on the biMAb-directed lytic activity of activated lymphocytes appeared to be minimal, although the same fluids were able to inhibit the lymphocyte activation phase (data not shown). Further confirmation of the stability of the reagent *in vivo* came from analysis in normal mice injected with M26.1 $F(ab')_2$, where it retained full T-cell targeting activity in the circulation, and clearance values were comparable to those obtained with parental and other MAb $F(ab')_2$ fragments (Fan *et al.*, 1993*b*; Van Dijk *et al.*, 1991).

To verify the *in vivo* anti-tumour activity of our reagent, we chose a preclinical survival model of nude mice using the $EGFR^+$ human ovarian carcinoma cell line IGROV1. This model was selected because the tumour remains localised to the peritoneum and can therefore be treated with local immunotherapy. Specific *in vivo* targeting therapy using *in vitro*-activated lymphocytes coupled with biMAb has been described (Nitta *et al.*, 1990; Bolhuis *et al.*, 1992), and other clinical trials with different biMAbs are currently underway. Consistent with data from preclinical and clinical studies, we find that mice treated with activated human PBLs coated with M26.1 $F(ab')_2$ survive significantly longer, although the treatment did not completely eradicate the implanted tumour. This failure probably reflects the high growth rate of the xenograft model which causes death more rapidly (mean survival time = 10.75 days) than in other preclinical models (Mezzanzanica *et al.*, 1991*a*; Renner *et al.*, 1994). More aggressive treatment might further improve survival rates and eventually lead to tumour cure.

This biMAb retargeting approach offers several advantages over others that utilise anti-EGFR MAbs therapeutically. Targeting of T cells by biMAb should allow a local release of inhibitory cytokines at the tumour site (Qian *et al.*, 1991) which might act on all cells within a tumour, including cells that were sterically inaccessible to targeted PBLs or that had lost antigen expression. In our experimental model, adverse effects on normal tissues would not be expected in mice because the biMAb does not bind to the mouse EGFR (Valota *et al.*, submitted) and to murine CD3 (Ferrini *et al.*, 1993). Since human epithelia express moderate/low EGFR levels (Rodriguez *et al.*, 1991; Banks-Schlegel *et al.*, 1986) the systemic use of anti-EGFR MAbs in humans must be considered with caution. However, bispecific M26.1 $F(ab')_2$ might be used as a targeting agent for local treatment of tumours such as gliomas or ovarian cancers that express variable levels of EGFR molecules, but whose surrounding accessible normal tissues are EGFR negative.

**Acknowledgements**
We thank E Luison for excellent technical help, M Azzini for photographic reproduction and D Labadini for manuscript preparation. This work was partially supported by grants from CNR-ACRO and AIRC.

**References**

BANKS-SCHLEGEL SP AND QUINTERO J. (1986). Human esophageal carcinoma cells have fewer, but higher affinity epidermal growth factor receptors. *J. Biol. Chem.*, **261**, 4359–4362.

BASELGA J, NORTON L, MASUI H, PANDIELLA A, COPLAN K, MILLER WH JR AND MENDELSOHN J. (1993). Antitumor effects of doxorubicin in combination with anti-epidermal growth factor receptor monoclonal antibodies. *J. Natl Cancer Inst.*, **85**, 1327–1333.

BENDER H, TAKAHASHI H, ADACHI K, BELSER P, LIANG S, PREWETT M, SCHRAPPE M, SUTTER A, RODECK U AND HERLYN D. (1992). Immunotherapy of human glioma xenografts with unlabeled, $^{131}I$-, or $^{125}I$-labeled monoclonal antibody 425 to epidermal growth factor receptor. *Cancer Res.*, **52**, 121–126.

BEUN GDM, VAN DE VELDE CJH AND FLEUREN GJ. (1994). T-cell based cancer immunotherapy. Direct or redirected tumor-cell recognition. *Immunol. Today*, **15**, 11–15.

BOLHUIS RLH, LAMERS CHJ, GOEY HS, EGGERMONT AMM, TRIMBOS JB, STOTER G, LANZAVECCHIA A, DI RE E, MIOTTI S, RASPAGLIESI F, RIVOLTINI L AND COLNAGHI MI. (1992). Adoptive immunotherapy of ovarian carcinoma with Bs-MAb targeted lymphocytes. A multicenter study. *Int. J. Cancer*, **7**, 78–81.

BRADY LW, MIYAMOTO C, WOO DV, RACKOVER M, EMRICH J, BENDER H, DADPARVAR S, STEPLEWSKI Z, KOPROWSKI H, BLACK P, LAZZARO B, NAIR S, MCCORMACK T, NIEVES J, MORABITO M AND ESHLEMAN J. (1992). Malignant astrocytomas treated with iodine-125 labeled monoclonal antibody 425 against epidermal growth factor receptor: A phase II trial. *Int. J. Radiat. Oncol. Biol. Phys.*, **22**, 225–230.

CHAFFANET M, CHAUVIN C, LAINÉ M, BERGER F, CHÉDIN M, ROST N, NISSOU M-F AND BENABID AL. (1992). EGF receptor amplification and expression in human brain tumours. *Eur. J. Cancer*, **28**, 11–17.

DIVGI CR, WELT S, KRIS M, REAL FX, YEH SDJ, GRALLA R, MERCHANT B, SCHWEIGHART S, UNGER M, LARSON SM AND MENDELSOHN J. (1991). Phase I and imaging trial of indium-111-labeled anti-epidermal growth factor receptor monoclonal antibody 225 in patients with squamous cell lung carcinoma. *J Natl Cancer Inst.*, **83**, 97–104.

FAN Z, BASELGA J, MASUI H AND MENDELSOHN J. (1993*a*). Antitumor effect of anti-epidermal growth factor receptor monoclonal antibodies plus *cis*-diamminedichloroplatinum on well established A431 cell xenografts. *Cancer Res.*, **53**, 4637–4642.

FAN Z, MASUI H, ATLAS I AND MENDELSOHN J. (1993*b*). Blockade of epidermal growth factor receptor function by bivalent and monovalent fragments of 225 anti-epidermal growth factor receptor monoclonal antibodies. *Cancer Res.*, **53**, 4322–4328.

FANGER MW, SHEN L, GRAZIANO RF AND GUYRE PM. (1989). Cytotoxicity mediated by human Fc receptors for IgG. *Immunol. Today*, **10**, 92–99.

FANGER MW, MORGANELLI PM AND GUYRE PM. (1992). Bispecific antibodies. *Crit. Rev. Immunol.*, **12**, 101–124.

FERRINI S, PRIGIONE I, MAMMOLITI S, COLNAGHI MI, MÉNARD S AND MORETTA A. (1989). Retargeting of T-cell-receptor gamma/delta + lymphocytes against tumor cells by bispecific monoclonal antibodies. Induction of cytolytic activity and lymphokine production. *Int. J. Cancer*, **4**, 53–55.

FERRINI S, CAMBIAGGI A, SFORZINI S, MARCIANO S, CANEVARI S, MEZZANZANICA D, COLNAGHI MI, GROSSI CE AND MORETTA L. (1993). Targeting of T lymphocytes against EGF-Receptor + tumor cells by bispecific monoclonal antibodies. Requirement of CD3 molecule cross-linking for T-cell activation. *Int. J. Cancer*, **55**, 931–937.

FOX SB, SMITH K, HOLLYER J, GREENALL M, HASTRICH D AND HARRIS AL. (1994). The epidermal growth factor receptor as a prognostic marker. Results of 370 patients and review of 3009 patients. *Breast Cancer Res. Treat.*, **29**, 41–49.

GILL GN, KAWAMOTO T, COCHET C, LE A, SATO JD, MASUI H, MCLEOD C AND MENDELSOHN J. (1984). Monoclonal anti-epidermal growth factor receptor antibodies which are inhibitors of epidermal growth factor binding and antagonists of epidermal growth factor-stimulated tyrosine protein kinase activity. *J. Biol. Chem.*, **259**, 7755–7760.

GULLICK WJ. (1991). Prevalence of aberrant expression of the epidermal growth factor receptor in human cancers. *Br. Med. Bull.*, **47**, 87–98.

HENDLER FJ, SHUM-SIU A, OECHSLI M, NANU L, RICHARDS CS AND OZANNE BW. (1989). Increased EGF-R1 binding predicts a poor survival in squamous tumors. *Cancer Cells*, **7**, 347–351.

HIRTE H AND CLARK DA. (1991). Generation of lymphokine-activated killer cells in human ovarian carcinoma ascitic fluid: Identification of transforming growth factor-b as a suppressive factor. *Cancer Immunol. Immunother.*, **32**, 296–302.

KALOFONOS HP, PAWLIKOWSKA TR, HEMINGWAY A, COURTENAY-LUCK N, DHOKIA B, SNOOK D, SIVOLAPENKO GB, HOOKER GR, MCKENZIE CG, LAVENDER PJ, THOMAS DGT AND EPENETOS AA. (1989). Antibody guided diagnosis and therapy of brain gliomas using radiolabeled monoclonal antibodies against epidermal growth factor receptor and placental alkaline phosphatase. *J. Nucl. Med.*, **30**, 1636–1645.

LIBERMANN TA, NUSBAUM HR, RAZON N, KRIS R, LAX I, SOREQ H, WHITTLE N, WATERFIELD MD, ULLRICH A AND SCHLESSINGER J. (1985). Amplification, enhanced expression and possible rearrangement of EGF receptor gene in primary human brain tumours of glial origin. *Nature*, **313**, 144–147.

MARCHALONIS JJ. (1969). An enzymatic method for the trace iodination of immunoglobulin and other proteins. *Biochem. J.*, **113**, 299–305.

MARIANI M, CAMAGNA M, TARDINI L AND SECCAMANI E. (1991). A new enzymatic method to obtain high-yield F(ab)$_2$ suitable for clinical use from mouse IgG1. *Mol. Immunol.*, **28**, 69–77.

MENDELSOHN J. (1990). The epidermal growth factor receptor as a target for therapy with antireceptor monoclonal antibodies. *Cancer Biol.*, **1**, 339–344.

MEZZANZANICA D, GARRIDO MA, NEBLOCK DS, DADDONA PE, ANDREW SM, ZURAWSKI VR JR, SEGAL DM AND WUNDERLICH JR. (1991*a*). Human T-lymphocytes targeted against an established human ovarian carcinoma with a bispecific F(ab')$_2$ antibody prolong host survival in a murine xenograft model. *Cancer Res.*, **51**, 5716–5721.

MEZZANZANICA D, CANEVARI S AND COLNAGHI MI. (1991*b*). Retargeting of human lymphocytes against human ovarian carcinoma cells by bispecific antibodies: from laboratory to clinic. *Int. J. Clin. Lab. Res.*, **21**, 159–164.

NITTA T, SATO K, YAGITA H, OKUMURA K AND ISHI S. (1990). Preliminary trial of specific targeting therapy against malignant glioma. *Lancet*, **335**, 368–376.

PARHAM P. (1983). On the fragmentation of monoclonal IgG1, IgG2a, and IgG2b from balb/c mice. *J. Immunol.*, **131**, 2895.

PUPA SM, CANEVARI S, FONTANELLI R, MÉNARD S, MEZZANZANICA D, LANZAVECCHIA A AND COLNAGHI MI. (1988). Activation of mononuclear cells to be used for hybrid monoclonal antibody-induced lysis of human ovarian carcinoma cells. *Int. J. Cancer*, **42**, 455–459.

QIAN JH, TITUS JA, ANDREW SM, MEZZANZANICA D, GARRIDO MA, WUNDERLICH JR AND SEGAL DM. (1991). Human peripheral blood lymphocytes targeted with bispecific antibodies release cytokines that are essential for inhibiting tumor growth. *J. Immunol.*, **146**, 3250–3256.

RENNER C, JUNG W, SAHIN U, DENFELD R, POHL C, TRÜMPER L, HARTMANN F, DIEHL V, VAN LIER R AND PFREUNDSCHUH M. (1994). Cure of xenografted human tumors by bispecific monoclonal antibodies and human T cells. *Science*, **264**, 833–835.

RODECK U, HERLYN M, HERLYN D, MOLTHOFF C, ATKINSON B, VARELLO M, STEPLEWSKI Z AND KOPROWSKI H. (1987). Tumor growth modulation by a monoclonal antibody to the epidermal growth factor receptor:immunologically mediated and effector cell-independent effects. *Cancer Res.*, **47**, 3692–3696.

RODRIGUEZ GC, BERCHUCK A, WHITAKER RS, SCHLOSSMAN D, CLARKE-PEARSON DL AND BAST RC JR. (1991). Epidermal growth factor receptor expression in normal ovarian epithelium and ovarian cancer. II. Relationship between receptor expression and response to epidermal growth factor. *Am. J. Obstet. Gynecol.*, **164**, 745–750.

RUFFINI PA, RIVOLTINI L, SILVANI A, BOIARDI A AND PARMIANI G. (1993). Factors including transforming growth factor b, released in the glioblastoma residual cavity, impair activity of adherent lymphokine-activated killer cells. *Cancer Immunol. Immunother.*, **36**, 409–416.

SEGAL DM, GARRIDO MA, PEREZ P, TITUS JA, WINKLER DA, RING DB, KAUBISCH A AND WUNDERLICH JR. (1988). Targeted cytotoxic cells as a novel form of cancer immunotherapy. *Mol. Immunol.*, **25**, 1099.

SHARKEY RM, NATALE A, GOLDENBERG DM AND MATTES MJ. (1991). Rapid blood clearance of immunoglobulin g2a and immunoglobulin g2b in nude mice. *Cancer Res.*, **51**, 3102–3107.

VALOTA O, CANEVARI S, TOSI E, ADOBATI E, CASALINI P, PEREZ P AND COLNAGHI MI. (1995). Anti-idiotypic response to anti-growth factor receptor monoclonal antibodies. *Eur. J. Cancer*, (submitted).

VAN DIJK J, ZEGVELD ST, FLEUREN GJ AND WARNAAR SO. (1991). Localization of monoclonal antibody G250 and bispecific monoclonal antibody CD3/G250 in human renal-cell carcinoma xenografts: Relative effects of size and affinity. *Int. J. Cancer*, **48**, 738–743.

WAHL SM, MCCARTNEY-FRANCIS N AND MERGENHAGEN SE. (1989). Inflammatory and immunomodulatory roles of TGF-B. *Immunol. Today*, **10**, 258–261.

WONG AJ, RUPPERT JM, BIGNER SH, GRZESCHNIK CH, HUMPHREY PA, BIGNER DS AND VOGELSTEIN B. (1992). Structural alterations of the epidermal growth factor receptor gene in human gliomas. *Proc. Natl Acad Sci. USA*, **89**, 2965–2969.